# Physical Activity Across Adulthood in Relation to Fat and Lean Body Mass in Early Old Age: Findings From the Medical Research Council National Survey of Health and Development, 1946–2010

**DOI:** 10.1093/aje/kwu033

**Published:** 2014-04-09

**Authors:** David Bann, Diana Kuh, Andrew K. Wills, Judith Adams, Soren Brage, Rachel Cooper, on behalf of the National Survey of Health and Development scientific and data collection team

**Keywords:** body fat distribution, motor activity, obesity, sarcopenia, sedentary lifestyle, skeletal muscle

## Abstract

Fat and lean body mass have important implications for health and physical functioning in older age, and physical activity is purported to be an important modifiable determinant. However, our evidence-based understanding of its role is limited. We examined the associations of physical activity, assessed both by self-report (using data on leisure time physical activity (LTPA) collected on 4 occasions over a 28-year period) and objectively (using 5-day heart rate and movement monitoring), with fat and lean mass at ages 60–64 years in 1,162 British participants from the Medical Research Council National Survey of Health and Development in 1946–2010. Higher objectively assessed physical activity energy expenditure (PAEE) at ages 60–64 years was associated with lower fat mass and android (abdominal):gynoid (hip) fat ratio (mean differences in fat mass per 1–standard deviation increase in PAEE were −0.79 kg/m^1.2^ in men (95% confidence interval: −1.08, −0.50) and −1.79 kg/m^1.2^ (95% confidence interval: −2.15, −1.42) in women). After adjustment for fat mass, higher PAEE was associated with higher appendicular lean mass. Both light and moderate-to-vigorous intensities of activity were associated with fat mass, and the latter was associated with lean mass. More frequent LTPA across adulthood was associated with lower fat mass (in women only) and higher appendicular lean mass (in both sexes, after adjustment for fat mass). These results support the promotion of LTPA across adulthood, as well as both light and moderate-to-vigorous intensities of activity among older adults.

Obesity is associated with increased morbidity and premature mortality risks (1, 2); as such, its prevention and prevalence reduction are important public health targets. Preventing low muscle mass in the population is also important given the roles of muscle in health and functioning (3, 4), particularly at older ages when age-related declines in muscle mass tend to occur (5, 6).

Although physical activity is likely to have a role in preventing obesity and in influencing muscle (lean) mass gain and maintenance, an evidence-based understanding of its role is limited because few observational studies have examined associations between free-living physical activity measures and direct measures of fat and/or lean mass in adulthood, and existing studies have produced inconsistent findings (7–16). In the Health, Aging, and Body Composition Study (*n =* 302), higher physical activity energy expenditure (PAEE) was cross-sectionally associated with greater lean mass in old age but was not associated with fat mass, nor with changes in lean or fat mass over 5 years (14). In contrast, in the Medical Research Council Ely Study (*n =* 739), higher PAEE was associated with greater gains in fat and lean mass over approximately 5.6 years in older middle-aged adults, but with greater declines in fat but not lean mass in younger middle-aged adults (7). Other cross-sectional studies found that higher physical activity is related to lower fat mass (11, 15) or found this association in men but not women (9) (or vice versa (10)). Existing studies also have important limitations that lead to uncertainty about the role of physical activity in influencing fat and lean mass. For example, many used single-sex samples (11, 12) or small sample sizes (*n* < 100) (8–10, 12), or they examined fat but not lean mass (9–11, 15, 16) (or vice versa (13)). Most used total physical activity estimates (7, 9, 10, 14) and did not examine intensities of activity. Although light activity and moderate-to-vigorous physical activity (MVPA) both contribute to total activity energy expenditure, MVPA may contribute more, suppress appetite (17), and lead to higher basal metabolic rate (18), leading to stronger inverse associations with fat mass. MVPA may also capture the types of activity that are important for lean mass, such as muscle-loading activities (19). Associations with lean mass could be confounded by fat mass; because of adaptive mechanisms, gains or losses in fat typically lead to respective gains or losses in lean mass (20, 21). Despite this, previous studies have not examined whether physical activity is associated with lean mass independently of fat mass. Finally, previous studies have used single measures of physical activity ascertained at 1 point in life, and none has examined whether there are cumulative benefits of physical activity across adulthood leading to lower fat and higher lean mass.

The objectives of this study were to examine the associations between physical activity and body composition in a large British birth cohort study. This study benefits from repeat prospective reports of leisure time physical activity (LTPA), objective measures of free-living physical activity of different intensities, and direct measures of body composition in early old age.

## METHODS

### Subjects

The Medical Research Council National Survey of Health and Development (NSHD) is a socially stratified sample of 5,362 singleton births that took place in 1 week of March 1946 in mainland Britain (22), with regular follow-up across life. Between 2006 and 2010 (at ages 60–64 years), 2,856 eligible study members (known to be alive and with British addresses) were invited for an assessment at 1 of 6 clinical research facilities or to be visited by a research nurse at home. Invitations were not sent to those who had died (*n =* 778), who were living abroad (*n =* 570), who had previously withdrawn from the study (*n =* 594), or who had been lost to follow-up (*n =* 564). Of those invited, 2,229 (78.0%) were assessed. Of these, 1,690 (75.8%) attended a clinical research facility, and the remaining 539 were seen at home (23). The study received multicenter research ethics committee approval, and informed consent was given by participants.

### Body composition measurement

During the visits to the clinical research facilities (not home visits), supine measurements of body composition were obtained using a QDR 4500 Discovery dual-energy x-ray absorptiometry scanner (Hologic Inc., Bedford, Massachusetts) with APEX, version 3.1, software (Hologic Inc.) as previously described (24). Outcome measures were whole body fat mass (in kg), android (abdominal):gynoid (hip) fat mass ratio (multiplied by 100), and appendicular lean mass (in kg) (*n =* 1,558). Height and weight were measured by trained nurses using standardized protocols (23).

### Physical activity

Objective measurements of physical activity were obtained using chest-worn Actiheart movement and heart rate monitors (CamNtech, Ltd., Papworth, United Kingdom), which are technically reliable and valid, as reported elsewhere (25). Acceleration and heart rate were measured in 30-second epochs for up to 5 consecutive days. Heart rate data were individually calibrated to account for between-individual differences in the relationship between physical activity intensity and heart rate (26) using data on heart rate response to incremental aerobic exercise (8-minute step test) (*n =* 853) or, when not available (*n =* 309), using group calibration (Web Appendix 1 available at http://aje.oxfordjournals.org/) adjusted to individual sleeping heart rate, age, sex and β-blocker use. These data were used in branched equation modeling (27) to estimate intensity and were summarized as total PAEE (in kJ/kg/day) and time spent in different intensities (relative to resting metabolic rate, 1 standard metabolic equivalent (MET)) as follows: sedentary (≤1.5 METs), light (1.5–3 METs), and MVPA (>3 METs)). These measures have been validated using indirect calorimetry and doubly labeled water in adults during experimental (26, 28) and free-living activities (29). None of these measures uses individual body weight in its calculation (26).

To ensure that physical activity estimates were reasonably accurate reflections of normal behavior, participants with less than 48 hours of free-living data were excluded from analyses (*n =* 24). All data were adjusted for wear time, estimated by both movement and heart rate monitoring, as well as for diurnal information bias. All heart rate and movement traces were visually inspected, and participants were excluded when the acceleration signal was corrupt or severe clinical irregularities prevented valid heart rate measurements for extended periods (*n =* 55).

LTPA measurements were obtained at ages 36, 43, 53, and 60–64 years during interviews with research nurses (30). At age 36 years, participants were asked how often in the previous month they participated in 27 different leisure time activities using a modified Minnesota LTPA questionnaire (31). At ages 43, 53, and 60–64 years, participants were asked how often in the previous month or 4-week period they participated in any sports, vigorous leisure activities, or exercises. At each age, participants were categorized as inactive (no participation), moderately active (1–4 times), or most active (≥5 times).

### Potential confounders

The following were selected a priori: indicators of socioeconomic position ascertained prior to physical activity assessment (paternal occupational class (when subject was 4 years of age) and own educational attainment (at age 26 years)) and the presence or absence of any long-term illness, health problem, or disability that limited activities (self-reported at age 60–64 years), as used in the 2001 England and Wales Census (32).

### Statistical analysis

Because taller individuals tend to have more fat and lean mass, height-adjusted indices were created by dividing fat and lean mass (in kg) by height (m)*^X^* (*X* = 1.2 for fat mass, and *X* = 2 for lean mass), where *X* was calculated so that the resulting index was not correlated with height (33). Linear regression was used to examine unadjusted associations between each physical activity measure and each outcome. PAEE was converted to sex-specific *z* scores to aid coefficient presentation. To investigate whether light physical activity and/or MVPA was independently associated with outcomes, we conducted additional mutually adjusted analyses. Because changes in fat mass in early- to mid-adulthood typically lead to respective changes in lean mass (20, 21), models with lean mass as the outcome were repeated with adjustment for fat mass. Models with all outcomes were repeated with adjustment for potential confounders.

Because previous studies have found sex differences in associations, all analyses were conducted separately by sex; sex differences were formally tested by including a sex interaction term. Deviation from linearity was assessed by visually inspecting scatter plots and by comparing models in which activity was modeled as a linear term and as a nonlinear term. Complete case analyses were used (*n =* 1,162 using objectively assessed measures; *n =* 1,211 using LTPA).

A second set of analyses was conducted to examine whether there was a cumulative association between LTPA across adulthood and fat and lean mass. First, associations between LTPA at ages 36, 43, and 53 years were additionally adjusted for LTPA at age 60–64 years. Second, a lifetime physical activity score was derived by summing LTPA at all 4 ages (coded at each age as 0 (inactive), 1 (moderately active), or 2 (most active)). This score (range, 0 (inactive at all 4 ages) to 8 (most active at all 4 ages)) was categorized into 4 groups of similar size (0–1, 2–3, 4–5, and 6–8), and associations with outcomes were examined using linear regression. Third, a structured modeling approach (30, 34) was used to examine whether models specifying accumulation of physical activity (allowing for varying effect sizes at each age) fitted the data as well as a more complex saturated model that contained parameters specifying accumulation, sensitive periods of activity at each age, and interactions between activity at each age. These models (Web Appendix 2) were compared using partial *F* tests, with larger *P* values indicating that the nested model fit the data as well as the saturated model.

### Sensitivity analyses

To examine whether the methods used to objectively assess PAEE affected our findings, we repeated the main analyses when 1) using group calibration for all participants (instead of individual calibration); 2) excluding participants taking β-blockers at ages 60–64 years; and 3) using average trunk acceleration (in m/second^2^) instead of PAEE.

## RESULTS

### Descriptive statistics

Men had higher PAEE and spent more time in MVPA than women at ages 60–64 years (Table 1). In both sexes, the majority of time was spent sedentary, and the least time was spent in MVPA. A total of 95% of participants wore their monitors for 4 or 5 days. More men than women reported participating in activities at ages 36 and 43 years, and in both sexes, participation at ages 60–64 years was lower than at all previous ages.

**Table 1.** Body Composition and Physical Activity Measures in the MRC National Survey of Health and Development, 1946–2010^a^

Measure	Men (*n =* 563)			Women (*n =* 599)			*P* Value^b^
	Mean (SD)	No.	%	Mean (SD)	No.	%	
Body composition at ages 60–64 years							
Fat mass index^c^	12.02 (3.63)			16.20 (4.97)			<0.001
Android fat mass, kg	2.47 (0.96)			2.34 (0.98)			0.02
Gynoid fat mass, kg	3.73 (1.00)			5.11 (1.41)			<0.001
Android (abdominal):gynoid (hip) fat mass ratio	65.16 (15.50)			44.93 (12.04)			<0.001
Appendicular lean mass index^d^	8.02 (0.95)			6.19 (0.87)			<0.001
Objectively measured physical activity at age 60–64 years							
Physical activity energy expenditure, kJ/kg/day	38.91 (15.54)			35.41 (13.50)			<0.001
Sedentary time (≤1.5 METs), hours/day	17.67 (2.15)			17.81 (2.04)			0.27
Light intensity (1.5–3 METs), hours/day	5.40 (1.76)			5.56 (1.73)			0.13
Moderate-to-vigorous intensity (>3 METs), hours/day	0.73 (0.35–1.23)^e^			0.47 (0.24–0.86)^e^			<0.01
Self-reported leisure time physical activity							
At age 36 years							
Inactive		166	29.17		227	35.36	
Moderately active		161	28.3		180	28.04	
Most active		242	42.53		235	36.6	0.04
At age 43 years							
Inactive		241	42.36		313	48.75	
Moderately active		143	25.13		168	26.17	
Most active		185	32.51		161	25.08	0.01
At age 53 years							
Inactive		226	39.72		274	42.68	
Moderately active		138	24.25		132	20.56	
Most active		205	36.03		236	36.76	0.28
At ages 60–64 years							
Inactive		345	60.63		365	56.85	
Moderately active		86	15.11		114	17.76	
Most active		138	24.25		163	25.39	0.34

Abbreviations: MET, metabolic equivalent; MRC, Medical Research Council; SD, standard deviation.

^a^ Analyses restricted to those with valid data for physical activity measures, paternal occupational class, own educational attainment, long-term limiting illness or disability, and all body composition outcomes; activity at each age was coded as inactive (no participation), moderately active (participated 1–4 times) and most active (participated ≥5 times), in the previous month (at age 36 years) or in the previous month or 4-week period (at ages 43, 53, and 60–64 years).

^b^ Comparison of sexes, using *t* tests or χ^2^ test, as appropriate.

^c^ Fat mass index is calculated as weight (kg)/height (m)^1.2^.

^d^ Appendicular lean mass index is calculated as appendicular lean mass (kg)/height (m)^2^.

^e^ Median (interquartile range) presented because of right skew (*P* value derived using the Mann-Whitney *U* test).

### Objectively measured physical activity and body composition

Higher PAEE at ages 60–64 years was associated with lower fat mass and android:gynoid ratio at ages 60–64 years (Table 2). Associations with fat mass were stronger in women than in men. Higher levels of light physical activity and MVPA were associated with lower fat mass and android:gynoid ratio more strongly in women than in men (Table 3). Among women, these associations were independent of each other; among men, associations with MVPA remained, whereas associations with light intensity physical activity were largely attenuated (Web Table 1). Light physical activity and MVPA were positively correlated in both men (*r* = 0.33) and women (*r* = 0.41).

**Table 2.** Mean Differences in Body Composition Outcomes at Ages 60–64 Years Per 1–Standard Deviation Increase in Total Physical Activity Energy Expenditure (in kJ/kg/day) at 60–64 Years, the MRC National Survey of Health and Development, 1946–2010^a^

Outcome by Sex	Unadjusted				Adjusted^b^			
	β	95% CI	*P* Value	*P* Value^c^	β	95% CI	*P* Value	*P* Value^c^
Men (*n =* 563)								
Fat mass index^d^	−0.79	−1.08, −0.50	<0.001	<0.001	−0.76	−1.05, −0.46	<0.001	<0.001
Android (abdominal):gynoid (hip) fat mass ratio	−1.88	−3.14, −0.62	<0.01	0.63	−1.84	−3.11, −0.58	<0.01	0.76
Appendicular lean mass index^e^	−0.03	−0.11, 0.05	0.45	0.04	−0.03	−0.10, 0.05	0.53	0.04
Appendicular lean mass index^f^	0.07	0.01, 0.14	0.03	0.68	0.08	0.01, 0.15	0.03	0.80
Women (*n =* 599)								
Fat mass index	−1.79	−2.15, −1.42	<0.001		−1.65	−2.02, −1.29	<0.001	
Android:gynoid fat mass ratio	−2.26	−3.19, −1.34	<0.001		−2.02	−2.96, −1.08	<0.001	
Appendicular lean mass index	−0.14	−0.20, −0.07	<0.001		−0.13	−0.20, −0.06	<0.001	
Appendicular lean mass index^f^	0.08	0.02, 0.13	<0.01		0.07	0.02, 0.13	<0.01	

Abbreviations: CI, confidence interval; MRC, Medical Research Council.

^a^ All analyses restricted to those with valid data for paternal occupational class, own educational attainment, long-term limiting illness or disability, and all body composition outcomes.

^b^ Adjusted for the following potential confounders: paternal occupational class when subject was 4 years of age, own educational attainment at age 26 years, and long-term limiting illness or disability at ages 60–64 years.

^c^ *P* value for sex interaction term.

^d^ Fat mass index is calculated as weight (kg)/height (m)^1.2^.

^e^ Appendicular lean mass index is calculated as appendicular lean mass (kg)/height (m)^2^.

^f^ Adjusted for fat mass index.

**Table 3.** Mean Differences in Body Composition Outcomes Per 1-Hour Increase in Time Spent Sedentary and in Light and Moderate-to-Vigorous Intensities of Physical Activity at Ages 60–64 Years, the MRC National Survey of Health and Development, 1946–2010^a^

Outcome Model by Sex	Sedentary^b^				Light Activity^c^				Moderate-to-Vigorous Activity^d^			
	β	95% CI	*P* Value	*P* Value^e^	β	95% CI	*P* Value	*P* Value^e^	β	95% CI	*P* Value	*P* Value^e^
Men (*n* = 563)												
Fat mass index^f^	0.28	0.14, 0.41	<0.001	<0.001	−0.22	−0.39, −0.05	0.01	<0.001	−0.97	−1.35, −0.60	<0.001	<0.001
Android (abdominal):gynoid (hip) fat mass ratio	0.50	−0.09, 1.10	0.10	0.19	−0.22	−0.95, 0.51	0.56	0.11	−2.69	−4.31, −1.07	<0.01	0.34
Appendicular lean mass index^g^	0.01	−0.03, 0.04	0.72	0.01	0.00	−0.05, 0.04	0.99	<0.01	−0.05	−0.15, 0.05	0.35	0.11
Appendicular lean mass index^h^	−0.03	−0.06, 0.00	0.07	0.77	0.03	−0.01, 0.07	0.16	0.91	0.08	−0.01, 0.17	0.07	0.27
Women (*n =* 599)												
Fat mass index	0.85	0.67, 1.03	<0.001		−0.89	−1.11, −0.67	<0.001		−2.60	−3.26, −1.94	<0.001	
Android:gynoid ratio	1.00	0.54, 1.47	<0.001		−0.96	−1.52, −0.41	<0.001		−3.85	−5.50, −2.20	<0.001	
Appendicular lean mass index	0.07	0.04, 0.11	<0.001		−0.08	−0.12, −0.04	<0.001		−0.18	−0.30, −0.06	<0.01	
Appendicular lean mass index^h^	−0.03	−0.06, 0.00	0.03		0.03	−0.01, 0.06	0.12		0.14	0.04, 0.23	<0.01	

Abbreviations: CI, confidence interval; MRC, Medical Research Council.

^a^ Analyses restricted to those with valid data for physical activity measures, paternal occupational class when subject was 4 years of age, own educational attainment at age 26 years, long-term limiting illness or disability at ages 60–64 years, and all body composition outcomes.

^b^ Sedentary, ≤1.5 metabolic equivalents

^c^ Light activity, 1.5–3 metabolic equivalents

^d^ Moderate-to-vigorous activity, >3 metabolic equivalents.

^e^ *P* value for sex interaction term.

^f^ Fat mass index is calculated as weight (kg)/height (m)^1.2^.

^g^ Appendicular lean mass index is calculated as appendicular lean mass (kg)/height (m)^2^.

^h^ Adjusted for fat mass index.

In unadjusted analyses, higher PAEE at ages 60–64 years was associated with lower appendicular lean mass in women, but after adjustment for fat mass, higher PAEE was associated with higher appendicular lean mass in both sexes (Table 2). Higher MVPA was associated with higher appendicular lean mass after adjustment for fat mass, whereas light intensity activity was not (Table 3 and Web Table 1).

Associations between sedentary time and outcomes mirrored associations with PAEE (Table 3); greater sedentary time was associated with higher fat mass, higher android:gynoid ratio, and lower appendicular lean mass (after adjustment for fat mass). Associations were similar after adjustment for potential confounders (Table 2 and Web Table 2).

### Self-reported LTPA across adulthood and body composition

**Figure 1.**
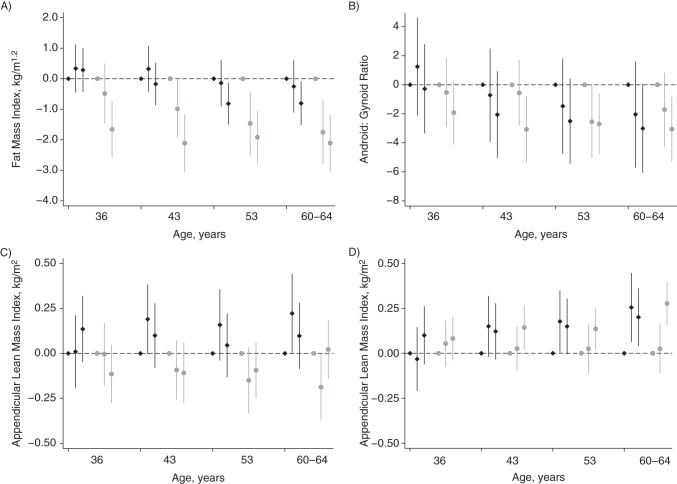
Mean difference in body composition outcomes at ages 60–64 years in those who were moderately and most active (compared with inactive) at ages 36, 43, 53, and 60–64 years in the Medical Research Council National Survey of Health and Development, United Kingdom, 1946–2010. A) Fat mass index (kg/m^1.2^), B) android (abdominal):gynoid (hip) fat mass ratio, C) appendicular lean mass (kg/m^2^), D) appendicular lean mass index (kg/m^2^) adjusted for fat mass index (kg/m^1.2^). The point estimates show, from left to right, those who were inactive, moderately active, and most active at each age. Men, black diamonds; women, gray circles. Activity at each age was coded as inactive (no participation; reference category), moderately active (participated 1–4 times), or most active (participated ≥5 times) in the previous month (at age 36 years) or in the previous month or 4-week period (at ages 43, 53, and 60–64 years). Samples included 569 men and 642 women. *P* for sex interaction term < 0.1 in all age groups in A and C and in the group aged 60–64 years in D. Bars, 95% confidence intervals.

Women who reported more LTPA across adulthood (at ages 36, 43, 53, and 60–64 years) tended to have lower fat mass at age 60–64 years, whereas associations (albeit weaker) were found only with LTPA at ages 53 and 60–64 years in men (Figure 1). Patterns of associations with android:gynoid ratio were similar but weaker.

There was some evidence that men who were active at each age had higher appendicular lean mass, whereas women had lower appendicular lean mass (Figure 1). After adjustment for fat mass, higher LTPA at each age was associated with higher appendicular lean mass in both sexes; these associations were weak at age 36 years. Associations between LTPA earlier in adulthood (at ages 36, 43, and 53 years) and outcomes were similar after adjustment for activity levels at ages 60–64 years (results available on request).

Higher lifetime physical activity score was associated with lower fat mass in women but not in men (Table 4), suggesting that, in women, there were cumulative benefits of LTPA across adulthood leading to lower fat mass. Results of life-course model comparisons supported this; models specifying cumulative effects of physical activity fitted the data as well as the saturated model (*P* = 0.70). In both sexes, higher lifetime physical activity was associated with lower android:gynoid ratio (in men, *P* = 0.30; in women, *P* = 0.51) and higher appendicular lean mass after adjustment for fat mass (in men, *P* = 0.08; in women, *P* = 0.10). These associations were similar after adjustment for potential confounders (Table 4).

**Table 4.** Mean Differences in Body Composition Outcomes at Ages 60–64 Years by Lifetime Physical Activity Score^a^, the MRC National Survey of Health and Development, 1946–2010

Lifetime Activity Score by Sex	No.	%	Fat Mass Index^b^		Android (Abdominal):Gynoid (Hip) Fat Mass Ratio		Appendicular Lean Mass Index^c^		Appendicular Lean Mass Index Adjusted for Fat Mass Index	
			Mean Difference	95% CI	Mean Difference	95% CI	Mean Difference	95% CI	Mean Difference	95% CI
Men										
** **Unadjusted										
0–1	126	22.14	0	Referent	0	Referent	0.00	Referent		Referent
2–3	150	26.36	0.57	−0.28, 1.43	−1.39	−5.07, 2.28	0.04	−0.19, 0.26	−0.04	−0.23, 0.15
4–5	153	26.89	0.8	−0.05, 1.65	−0.59	−4.24, 3.07	0.18	−0.04, 0.40	0.07	−0.12, 0.27
6–8	140	24.60	−0.64	−1.50, 0.23	−4.68	−8.41, −0.95	0.14	−0.09, 0.36	0.22	0.02, 0.42
*P*_trend_			0.20^d^		0.03		0.12		0.01	
*P* value^e^			<0.001		0.93		0.2		0.7	
Adjusted^f^										
0–1	126	22.14	0.00	Referent	0.00	Referent	0	Referent	0	Referent
2–3	150	26.36	0.81	−0.04, 1.66	−0.59	−4.25, 3.06	0.06	−0.17, 0.28	−0.05	−0.24, 0.15
4–5	153	26.89	1.18	0.32, 2.04	0.87	−2.83, 4.57	0.26	0.03, 0.48	0.1	−0.09, 0.30
6–8	140	24.60	−0.2	−1.08, 0.68	−2.94	−6.72, 0.84	0.22	−0.01, 0.45	0.24	0.04, 0.44
*P*_trend_			0.75^d^		0.22		0.02		<0.01	
*P* value^e^			<0.001		0.94		0.17		0.75	
Women										
Unadjusted										
0–1	153	23.83	0	Referent	0	Referent	0	Referent	0	Referent
2–3	197	30.69	−0.75	−1.80, 0.30	−2.05	−4.60, 0.50	−0.15	−0.34, 0.04	−0.06	−0.20, 0.08
4–5	151	23.52	−2.18	−3.30, −1.06	−1.9	−4.62, 0.81	−0.09	−0.29, 0.11	0.17	0.03, 0.32
6–8	141	21.96	−2.94	−4.08, −1.80	−4.72	−7.48, −1.95	−0.16	−0.37, 0.04	0.19	0.04, 0.34
*P*_trend_			<0.001		<0.01		0.2		<0.01^d^	
Adjusted^f^										
0–1	153	23.83	0	Referent	0	Referent	0	Referent	0	Referent
2–3	197	30.69	−0.51	−1.58, 0.55	−1.97	−4.56, 0.62	−0.15	−0.34, 0.04	−0.09	−0.23, 0.05
4–5	151	23.52	−1.89	−3.05, −0.74	−1.37	−4.18, 1.44	−0.11	−0.32, 0.09	0.12	−0.03, 0.27
6–8	141	21.96	−2.55	−3.72, −1.37	−4.1	−6.96, −1.24	−0.17	−0.38, 0.04	0.14	−0.02, 0.30
*P*_trend_			<0.001		0.01		0.17		0.01^d^	

Abbreviations: CI, confidence interval; MRC, Medical Research Council.

^a^ Lifetime physical activity score derived by adding the physical activity measures at ages 36, 43, 53, and 60–64 years, from none-lowest (0–1) to highest (6–8) activity; activity at each age was coded as 0 for inactive (no participation), 1 for moderately active (participated 1–4 times), and 2 for most active (participated ≥5 times) in the previous month (at age 36 years) or in the previous month or 4-week period (at ages 43, 53, and 60–64 years).

^b^ Fat mass index is calculated as weight (kg)/height (m)^1.2^.

^c^ Appendicular lean mass index is calculated as appendicular lean mass (kg)/height (m)^2^.

^d^ Evidence for departure from linearity (*P* < 0.05).

^e^ *P* value for sex interaction term.

^f^ Paternal occupational class when subject was 4 years of age, own educational attainment at age 26 years, and long-term limiting illness or disability at ages 60–64 years.

### Sensitivity analyses

Associations between objectively assessed physical activity measures and outcomes were similar when group equations were used for all participants and restricted to those not taking β-blockers at ages 60–64 years (results available on request). When we used average trunk acceleration instead of PAEE, associations with fat mass and android:gynoid ratio were similar, but associations with appendicular lean mass were weaker (Web Table 3).

## DISCUSSION

### Main findings

Higher levels of PAEE were associated with lower fat mass and android:gynoid ratio at ages 60–64 years in both sexes. In addition, higher PAEE was associated with higher appendicular lean mass (after adjustment for fat mass), and this was explained by variations in MVPA. There was evidence for cumulative benefits of participating in LTPA across adulthood (at ages 36, 43, 53, and 60–64 years) for lower fat mass (in women) and higher appendicular lean mass (in both sexes, after adjustment for fat mass).

### Comparison with previous studies

Findings from this study add to those of the few studies that have used objective measures of total physical activity in relation to fat and/or lean mass in later adulthood and produced inconsistent findings (7–16). The use of a larger sample size than previous studies may have contributed to results in the expected directions, with higher activity associated with lower fat and higher lean mass. This study also used a cohort assessed across a narrow age band and individually calibrated heart rate and movement monitoring data, which may have contributed to the results we found. We extend previous findings by examining 1) associations with both total physical activity and time spent at different intensities of activity, 2) whether associations with lean mass were independent of fat mass, and 3) whether LTPA across adulthood has cumulative benefits for fat and lean mass. These findings are consistent with those of previous NSHD studies, which found that higher LTPA was associated with lower obesity risk in women at age 36 years (35) and greater physical performance at age 53 years (30).

### Explanation of findings

Higher levels of both light physical activity and MVPA would independently contribute to total energy expenditure and, by shifting energy balance toward the negative, would be expected to lead to lower fat mass, especially abdominal fat (36). MVPA may have captured the types of activities that are beneficial for preservation or gains in muscle mass, such as resistance exercises (19), leading to associations with lean mass not found with light intensity activity. However, few participants in this sample reported exercising with weights (13% at ages 60–64 years), suggesting that the associations were driven by a broader range of activities, such as walking and sports participation (37), that require muscle strength and power to lift the weight of the body. Weaker associations with lean mass when using only the acceleration data may suggest that combined measures of accelerometry and heart rate are better able to capture activities that affect lean mass.

Associations between sedentary time and outcomes are likely to be wholly or partly explained by lower activity levels among more sedentary participants, because more sedentary time equates to less time spent in light physical activity or MVPA. Sedentary time could potentially affect body composition through other pathways, for example, through association with dietary behaviors or by physiological mechanisms initiated by inactivity (38, 39). This warrants future investigation because the strong correlations between sedentary time and activity measures prevented us from investigating this (correlations between sedentary time with light and MVPA in both sexes combined = −0.95 and −0.62, respectively). Because of the substitution of 1 activity intensity for another, the interpretation of associations with sedentary behavior, light activity, and MVPA are inextricably linked. In mutually adjusted models (Web Table 1) a 1-hour increase in light activity or MVPA reflects a 1-hour decrease in sedentary time (which includes sleep). Because wear time comprises these 3 components, examining the association of 1 activity component with an outcome while holding all others constant is not possible because of multicollinearity. Previous studies have included these components in the same model (40) but have omitted sleep time—such that an increase in any 1 component also reflects less sleep or nonwear time. Examination of the independence of associations with PAEE and activity intensities was not possible because of its strong correlation with both light activity and MVPA (*r* = 0.74 and 0.87, respectively, in both sexes combined).

Weaker associations between physical activity and fat mass in men could indicate that, among those with higher levels of physical activity, total energy intake was higher in men than in women. The activity measures we used may have also captured PAEE less effectively in men; at ages 60–64 years, men may have undertaken more peripheral body movements, which are less easily detected using trunk-mounted accelerometers, and at younger ages men who undertook more LTPA may have been more inactive in other domains (e.g., at work), resulting in no differences in total PAEE. Previous NSHD findings support this; LTPA was not associated with obesity in men at age 36 years but was in women (35) and was associated with more sedentary time at work (41).

Although we hypothesized that physical activity affected fat and lean mass, associations could be bidirectional. Psychological and physiological barriers may cause those with higher fat and lower lean mass to undertake less physical activity (42, 43), resulting in positive feedback loops. Additional analyses showed that associations between LTPA earlier in adulthood and outcomes were similar when adjusted for baseline body mass index (weight (kg)/height (m)^2^) (measured at age 36 years; Web Table 4), suggesting that findings were unlikely to be fully explained by reverse causation.

The indicators of socioeconomic position that we used are arguably distal indicators of other determinants of body composition such as diet (44, 45), which could operate as confounders. Although associations were similar after adjustment for these and other indicators such as own occupational class (results available on request), residual confounding cannot be ruled out, although it may be difficult to establish given inaccuracy in self-reported dietary intakes (46, 47). Physical activity may influence body composition by affecting appetite (17), further complicating the separation of physical activity and dietary influences on body composition.

### Strengths and limitations

Strengths of this study include the use of individually calibrated (27) combined heart rate and movement sensing, which has been demonstrated to produce more precise physical activity estimates in controlled settings than single measures of acceleration or heart rate (25), and the relation of these exposures to precise measurements of body composition. The repeated prospective measurements of LTPA across adulthood are also advantageous and enabled investigation of the cumulative associations of LTPA. The NSHD has a relatively large sample size of both sexes with prospectively ascertained measurements of potential confounders. Although the NSHD study members who participated at ages 60–64 were broadly representative of the nonmigrant British population of a similar age (48), associations between physical activity and body composition have been found to differ by age (7). Further studies are therefore needed in younger and older cohorts.

The physical activity measures used across adulthood were limited to LTPA; other domains of activity may also be important for body composition and therefore warrant investigation. However, LTPA is likely to be an important modifiable target given the increasingly sedentary nature of occupations in the developed world and is likely to be easily and accurately recalled. The sedentary time measure we used did not distinguish between time spent asleep or awake; because sleep duration may affect energy intake and expenditure (49), this warrants future investigation.

Loss to follow-up may have introduced bias. Less physically active participants with higher body mass index values at age 53 years were less likely to attend reassessment at ages 60–64 years (48); this likely led to reduced power to detect associations between lower physical activity and higher fat mass.

Individual calibration of physical activity data was not possible for all participants because of missing step-test data. However, sensitivity analyses suggested that the type of calibration we used did not substantially affect findings. Finally, MVPA was right skewed, but results were similar after log transformation (+1); unadjusted regression coefficients were therefore presented to aid interpretation.

### Implications

Findings suggest that both light physical activity and MVPA may lead to lower fat mass and lower android:gynoid ratio. Although most previous studies have focussed on MVPA (50, 51), the benefits of light intensities of activity are particularly relevant for older adults; light activities may be less likely to lead to falls, are more feasibly undertaken by those with higher morbidity rates, and may be more readily modified than more intensive activities that older adults may find difficult to initiate and maintain (40, 52). Given the potential of substituting some of the highly prevalent sedentary time with light activity or MVPA, as well as the expected benefits of even small changes in fat and lean mass at the population level, the effect sizes in this study were considerable. For example, results suggest that a 2-hour/day increase in light intensity activity would equate to fat mass index scores that are 3.7% lower in men and 11.0% lower in women (Table 3).

The association between higher MVPA and higher lean mass suggests that research is required to identify the specific types of activities that benefit muscle mass and can be implemented safely and sustained over the long term among older adults. Further research is required to determine whether physical activity can attenuate the age-related decline in lean mass.

Evidence for a cumulative association between LTPA across adulthood and lower fat mass (in women) and higher lean mass (in both sexes) supports the promotion of activity across adulthood on the basis of persisting benefits. Future studies should also consider the influence of physical activity trajectories across adulthood.

### Conclusions

Higher objectively assessed PAEE was associated with lower fat mass, lower android:gynoid ratio, and higher lean mass (independently of fat mass) in early old age. More frequent LTPA across adulthood was associated with lower fat mass (in women) and higher appendicular lean mass (in both sexes, after adjustment for fat mass).

## Supplementary Material

Web Material
