# Supplementary material for: Physical Activity Across Adulthood in Relation to Fat and Lean Body Mass in Early Old Age: Findings From the Medical Research Council National Survey of Health and Development, 1946–2010
Source: Am J Epidemiol. 2014 Apr 9;179(10):1197–207. doi: 10.1093/aje/kwu033 (PMC4010186; doi:10.1093/aje/kwu033)
Supplement: Web Material [file supp_179_10_1197__index.html]

Physical Activity Across Adulthood in Relation to Fat and Lean Body Mass in Early Old Age: Findings From the Medical Research Council National Survey of Health and Development, 1946–2010 — Physical Activity Across Adulthood in Relation to Fat and Lean Body Mass in Early Old Age: Findings From the Medical Research Council National Survey of Health and Development, 1946–2010 — Web Material 

# Physical Activity Across Adulthood in Relation to Fat and Lean Body Mass in Early Old Age: Findings From the Medical Research Council National Survey of Health and Development, 1946–2010

## Web Material

Web Material

**Files in this Data Supplement:**

- Web Material - Docx file
