# Supplementary material for: Physical Activity Across Adulthood in Relation to Fat and Lean Body Mass in Early Old Age: Findings From the Medical Research Council National Survey of Health and Development, 1946–2010
Source: Am J Epidemiol. 2014 Apr 9;179(10):1197–207. doi: 10.1093/aje/kwu033 (PMC4010186; doi:10.1093/aje/kwu033)
Supplement: Web Material [file supp_kwu033_kwu033supp.docx]

Web Appendix 1. Formula used to calculate instantaneous physical activity energy expenditure (PAEE) [J/min/kg] from heart rate in 60–64 year old individuals without valid step test:

PAEE [J/min/kg] = (14.08 - 0.138 * age + 0.39 * sex + 0.0021 * SHR + 0.51 * betablocker) * HRaS + 0.94 * age + 5.41 * sex - 0.76 * SHR + 12.3 * betablocker - 84.1

Note: age in years, sex coded as 1 for men and 0 for women; betablocker coded as 1 for yes and 0 for no; HR=heart rate in beats per minute; SHR=sleeping HR; HRaS=HR above SHR in beats per minute. The equation is derived using step-calibrated slope (ß_i_) and intercept (α_i_) parameters from the 937 individuals with a valid step test; PAEE_i_ = ß_i_*HRaS + α_i._

Web Appendix 2. Models compared when using the structured life course models

| Fully saturated model:  E(Y)= α + β_1_Pa_36_ + β_2_Pa_43_ + β_3_Pa_53_ + β_4_Pa_63_ + θ_12_Pa_36_Pa_43_ + θ_23_Pa_43_Pa_53_ + θ_13_Pa_36_Pa_53_ + θ_14_Pa_36_Pa_63_ + θ_242_Pa_43_Pa_63_ + θ_34_Pa_53_Pa_63_ + θ_1234_Pa_36_Pa_43_Pa_53_Pa_63_  Compared with:  (1) Critical/sensitive period model  E(Y)= α + β_1_Pa_36_  constraints: β_2_= β_3_= β_4_=0; θ_12_=θ_23_=θ_13_=θ_14_=θ_24_=θ_34_=θ_1234_=0  (2) Accumulation model: summed score (assuming similar effect sizes at each age)  E(Y)= α +βΣjPaj  constraints: β_1_=β_2_=β_3_=β_4_; θ_12_=θ_23_=θ_13_=θ_14_=θ_24_=θ_34_=θ_1234_=0  (3) Accumulation model: mutually adjusted (allowing for differences in effect size at each age)  E(Y)= α + β_1_Pa_36_ + β_2_Pa_43_ + β_3_Pa_53_ + β_4_Pa_63_  constraints: β_1_≠β_2_≠β_3_≠β_4_; θ_12_=θ_23_=θ_13_=θ_14_=θ_24_=θ_34_=θ_1234_=0 |
| --- |

Notes: Pa=physical activity at age 36, 43, 53, and 63 (60–64) years.

Web Table 1. Mean differences in body composition outcomes at 60-64 years per 1 hour increase in time spent in light and moderate-vigorous physical activity at 60-64 years (mutually adjusted for each other)

a) Men (n=563)

|  | Light |  |  | Moderate-vigorous |  |  |
| --- | --- | --- | --- | --- | --- | --- |
| Outcome models | β (95% CI) | P | P# | β (95% CI) | P | P# |
| Fat mass index (kg/m^1.2^) | -0.08(-0.26, 0.09) | 0.35 | <0.001 | -0.91(-1.31, -0.51) | <0.001 | 0.03 |
| Android: gynoid ratio | 0.20(-0.57, 0.97) | 0.61 | 0.14 | -2.84(-4.56, -1.12) | <0.01 | 0.79 |
| Appendicular lean mass index (kg/m^2^) | 0.01(-0.04, 0.05) | 0.76 | 0.02 | -0.05(-0.16, 0.05) | 0.33 | 0.64 |
| Appendicular lean mass index (kg/m^2^),  adjusted for fat mass index | 0.02(-0.02, 0.06) | 0.38 | 0.87 | 0.07(-0.03, 0.16) | 0.16 | 0.32 |

b) Women (n=599)

|  | Light |  | Moderate-vigorous |  |
| --- | --- | --- | --- | --- |
| Outcome models | β (95% CI) | P | β (95% CI) | P |
| Fat mass index (kg/m^1.2^) | -0.65(-0.88, -0.41) | <0.001 | -1.80(-2.51, -1.10) | <0.001 |
| Android: gynoid ratio | -0.53(-1.13, 0.07) | 0.08 | -3.20(-5.00, -1.39) | <0.001 |
| Appendicular lean mass index (kg/m^2^) | -0.07(-0.11, -0.02) | <0.01 | -0.09(-0.22, 0.04) | 0.16 |
| Appendicular lean mass index (kg/m^2^),  adjusted for fat mass index | 0.01(-0.02, 0.04) | 0.53 | 0.12(0.02, 0.23) | 0.02 |

Notes: #P-value for sex interaction term; Light 1.5–3 METs; Moderate-vigorous>3 METs; analyses restricted to those with valid data for physical activity measures, paternal occupational class, own educational attainment, long term limiting illness or disability, and all body composition outcomes

Web Table 2. Mean differences in body composition outcomes at 60-64 years per 1 hour increase in time spent sedentary, in light and moderate-vigorous intensities of physical activity at 60-64 years, adjusted for potential confounders*

a) Men (n=563)

|  | Sedentary |  |  | Light |  |  | Moderate-vigorous |  |  |
| --- | --- | --- | --- | --- | --- | --- | --- | --- | --- |
| Outcome models | β (95% CI) | P | P# | β (95% CI) | P | P# | β (95% CI) | P | P# |
| Fat mass index (kg/m^1.2^) | 0.27(0.13, 0.41) | <0.001 | <0.001 | -0.22(-0.39, -0.05) | 0.01 | <0.001 | -0.90(-1.28, -0.53) | <0.001 | <0.001 |
| Android: gynoid ratio | 0.51(-0.09, 1.11) | 0.10 | 0.23 | -0.24(-0.97, 0.49) | 0.51 | 0.12 | -2.53(-4.13, -0.92) | <0.01 | 0.52 |
| Appen. lean mass index (kg/m^2^) | 0.01(-0.03, 0.04) | 0.71 | <0.01 | 0.00(-0.05, 0.04) | 0.91 | <0.01 | -0.04(-0.14, 0.06) | 0.45 | 0.12 |
| Appen. lean mass index (kg/m^2^),  adjusted for fat mass index | -0.03(-0.06, 0.00) | 0.08 | 0.88 | 0.03(-0.01, 0.07) | 0.20 | 1.00 | 0.08(-0.01, 0.17) | 0.07 | 0.36 |

b) Women (n=599)

|  | Sedentary |  | Light |  | Moderate-vigorous |  |
| --- | --- | --- | --- | --- | --- | --- |
| Outcome models | β (95% CI) | P | β (95% CI) | P | β (95% CI) | P |
| Fat mass index (kg/m^1.2^) | 0.80(0.61, 0.98) | <0.001 | -0.84(-1.06, -0.63) | <0.001 | -2.30(-2.97, -1.63) | <0.001 |
| Android: gynoid ratio | 0.91(0.44, 1.38) | <0.001 | -0.89(-1.44, -0.34) | <0.01 | -3.33(-5.01, -1.65) | <0.001 |
| Appen. lean mass index (kg/m^2^) | 0.07(0.03, 0.10) | <0.001 | -0.07(-0.11, -0.03) | <0.001 | -0.17(-0.29, -0.05) | <0.01 |
| Appen. lean mass index (kg/m^2^),  adjusted for fat mass index | -0.03(-0.06, 0.00) | 0.03 | 0.03(0.00, 0.06) | 0.08 | 0.11(0.02, 0.21) | 0.02 |

Notes: *paternal occupational class at 4 years, own educational attainment at 26 years, and long term limiting illness or disability at 60–64 years; #P-value for sex interaction term; Appen=appendicular; sedentary≤1.5 metabolic equivalent (METs); Light 1.5–3 METs; Moderate-vigorous>3 METs.

Web Table 3. Mean differences in body composition outcomes at 60-64 years per 1 standard deviation increase in average acceleration per day (m/s^2^) at 60-64 years

|  | Men (n=563) |  | Women (n=599) |  |  |
| --- | --- | --- | --- | --- | --- |
| Outcome models | β (95% CI) | P | β (95% CI) | P | P# |
| Fat mass index (kg/m^1.2^) | -0.86(-1.15, -0.57) | <0.001 | -1.39(-1.76, -1.01) | <0.001 | 0.03 |
| Android: gynoid fat mass ratio | -2.17(-3.43, -0.90) | <0.001 | -2.05(-2.99, -1.12) | <0.001 | 0.89 |
| Appendicular lean mass index (kg/m^2^) | -0.05(-0.13, 0.03) | 0.20 | -0.13(-0.19, -0.06) | <0.001 | 0.15 |
| Appendicular lean mass index (kg/m^2^), adjusted for fat mass index | 0.06(-0.01, 0.13) | 0.08 | 0.04(-0.02, 0.09) | 0.20 | 0.78 |

Note: #P-value for sex interaction term; analyses restricted to those with valid data for paternal occupational class at 4 years, own educational attainment at 26 years, and long term limiting illness or disability at 60–64 years, and all body composition outcomes

Web Table 4. Mean differences in body composition outcomes (95% confidence intervals) at 60-64 years by lifetime physical activity score, adjusting for body mass index (kg/m^2^) at 36 years

| Lifetime activity  score | N (%) | Fat mass  index (kg/m^1.2^) | Android: gynoid  ratio | Appendicular lean  mass index (kg/m^2^) | Appendicular lean  mass index  (kg/m^2^), adjusted for fat mass index (kg/m^1.2^) |
| --- | --- | --- | --- | --- | --- |
| Men |  |  |  |  |  |
| 0-1 | 125 (22.08) | 0.00 | 0.00 | 0.00 | 0.00 |
| 2-3 | 149 (26.33) | 0.41(-0.27, 1.09) | -1.83(-5.27, 1.61) | -0.02(-0.19, 0.14) | -0.04(-0.20, 0.13) |
| 4-5 | 153 (27.03) | 0.53(-0.15, 1.21) | -1.34(-4.76, 2.09) | 0.09(-0.07, 0.26) | 0.08(-0.09, 0.24) |
| 6-8 | 139 (24.56) | -0.57(-1.26, 0.13) | -4.57(-8.07, -1.08) | 0.14(-0.03, 0.31) | 0.16(-0.01, 0.33) |
| P(trend) |  | 0.14 | 0.02 | 0.04 | 0.02 |
| P# |  | 0.03 | 0.84 | 0.88 | 0.54 |
|  |  |  |  |  |  |
| Women |  |  |  |  |  |
| 0-1 | 152 (23.82) | 0.00 | 0.00 | 0.00 | 0.00 |
| 2-3 | 196 (30.72) | 0.11(-0.71, 0.94) | -0.93(-3.34, 1.49) | 0.00(-0.14, 0.15) | -0.01(-0.14, 0.12) |
| 4-5 | 151 (23.67) | -0.94(-1.82, -0.05) | -0.24(-2.82, 2.34) | 0.13(-0.03, 0.29) | 0.21(0.07, 0.35) |
| 6-8 | 139 (21.79) | -1.56(-2.47, -0.66) | -3.02(-5.66, -0.38) | 0.08(-0.08, 0.24) | 0.21(0.07, 0.35) |
| P(trend) |  | <0.001 | 0.05 | 0.14 | <0.001 |

Notes: #P-value for sex interaction term; *evidence for departure from linearity (P<0.05); lifetime physical activity score derived by adding the physical activity measures at 36, 43, 53 and 60-64 years, from none-lowest (0-1) to highest (6-8) activity; activity at each age was coded as 0 inactive (no participation), 1 moderately active (participated one to four times) and 2 most active (participated five or more times), in the previous month (36 years), per month (43 years) and in the previous 4 weeks (53 and 60-64 years); analyses restricted to those with valid data for paternal occupational class, own educational attainment, long term limiting illness or disability, and all body composition outcomes
